# *Staphylococcus pettenkoferi* Bacteremia in an American Intensive Care Unit

**DOI:** 10.1155/2021/5235691

**Published:** 2021-09-29

**Authors:** Cameron Strong, Michael Cosiano, Melanie Cabezas, J. W. Barwatt, L. Gayani Tillekeratne

**Affiliations:** ^1^Department of Medicine, Duke University School of Medicine, Durham, NC, USA; ^2^Department of Psychiatry, Duke University School of Medicine, Durham, NC, USA; ^3^Division of Infectious Diseases, Department of Medicine, Duke University School of Medicine, Durham, NC, USA

## Abstract

Coagulase-negative staphylococci (CoNS) are considered the most common cause of nosocomial bloodstream infections; yet, these species are frequently designated as contaminants in the absence of systemic signs and symptoms of infection. Immunocompromised patients or those with prosthetic devices are at increased risk for clinically significant bacteremia. With the advent of matrix-assisted laser desorption ionization-time of flight mass spectrometry (MALDI-TOF MS) in clinical practice, there has been improved specificity of CoNS isolate identification and further elucidation of underrecognized pathogenic species. *Staphylococcus pettenkoferi* was a novel CoNS species first identified in 2002 and thought to be misdiagnosed as other CoNS due to limitations in biochemical identification. There is increasing identification of *S. pettenkoferi* isolates; however, there are limited case reports of clinically significant *S. pettenkoferi* bacteremia and no reported cases within the United States. We present the first known case of *S. pettenkoferi* from an American intensive care unit.

## 1. Introduction

Coagulase-negative staphylococci (CoNS) are typical skin flora which, when found to be cultured in human blood samples, are usually attributed to blood culture contamination due to sample collection or handling. Criteria to differentiate sample contamination from true bacteremia are lacking [1, 2]. Clinical practice places importance on the collection and interpretation of multiple samples to both increase yield and reduce risk of applying clinical significance to cultures growing stowaway skin flora.

German clinicians [3] described a novel CoNS species isolated from two patients in 2002. Named in recognition of Bavarian chemist Max von Pettenkofer, *Staphylococcus pettenkoferi* joined a group of bacteria known to exist as skin flora but potentially portends pathogenicity against humans. A Canadian literature review [4] describes nine case reports of *S. pettenkoferi* true bacteremia globally and adds a tenth case observed in Canada. Their review does not describe any cases in the United States.

Here we describe a case of *S. pettenkoferi* causing true bacteremia in a United States intensive care unit. Opportunistic infection possibly occurred due to iatrogenic immune suppression. Additional literature review suggests that no other clinical reports of true bacteremia caused by this organism have been fully reported in the United States.

## 2. Case Presentation

The 73-year-old male patient presented to an emergency department with bilateral lower extremity weakness and abdominal pain. He was hospitalized and underwent extensive neurologic workup due to a rapidly progressive sensorimotor neuropathy. Diaphragmatic involvement and subsequent ventilator dependence required tracheostomy placement and prolonged hospitalization. The patient was determined to have an inflammatory neuropathy due to anti-neurofascin 155 antibodies. Treatment options for this pathology are limited and poorly defined, but theoretic benefit could be achieved via anti-CD20 biologic therapy with rituximab. A six-dose course was planned and initiated.

The hospitalization was complicated by voluminous respiratory secretions and three separate clinical syndromes consistent with ventilator-associated pneumonia. Despite aggressive nursing care, the patient developed a sacral wound which progressed to stage 3. Given need for multiple antibiotic and vasoactive infusions over the course of his stay, a peripherally inserted central catheter (PICC) was placed on hospital day fourteen. These infections created delays in biologic therapy for his inflammatory neuropathy. However, he was able to receive his second of six planned rituximab infusions on hospital day fifty-eight.

On hospital day sixty, the patient developed hypotension, hypoxia, and leukocytosis consistent with sepsis physiology. Two blood cultures were collected peripherally forty minutes apart as part of broad workup for decompensation. Neither was drawn from the PICC line. After twenty-one hours, the first culture resulted as Gram-positive cocci in clusters, and rapid multiplexed polymerase chain reaction (PCR) testing by the FilmArray blood culture identification panel (BioFire Diagnostics LLC, Salt Lake City, UT) suggested coagulase-negative staphylococcus with resistance due to *mecA*. No change in clinical management was made at that time, as the single blood culture demonstrating CoNS was suspected to represent sample contamination, and the patient was already being treated empirically with a regimen including vancomycin. However, within 72 hours of collection, both blood culture samples demonstrated growth with CoNS, which was identified as *S. pettenkoferi* by matrix-assisted laser desorption ionization-time of flight mass spectrometry (MALDI-TOF MS). Both isolates showed susceptibility to vancomycin by Microscan testing (Beckman Coulter, CA, USA; see Table 1).

The PICC line was discontinued on hospital day sixty-one with device tip culture also growing *S. pettenkoferi.* Serial blood cultures were persistently positive for *S. pettenkoferi* through hospital day sixty-three. He demonstrated clinical stability on vancomycin for treatment of *S. pettenkoferi* bacteremia, and culture clearance was achieved on hospital day sixty-five. Vancomycin was continued for a fourteen-day course from culture clearance and completed on hospital day seventy-nine.

Unfortunately, his six-dose rituximab schedule continued to be interrupted by recurrent sepsis secondary to *Proteus* bacteremia by urinary source. He ultimately completed four doses of his planned six-dose rituximab course in the ICU and was transferred to the general medicine ward. At the time of manuscript submission, he was beyond ninety days of hospitalization and was yet to experience any neurologic recovery.

## 3. Discussion

Given the complexity of our patient's presentation and multiple possible sources as drivers for sepsis, it was not immediately clear to what degree the presence of *S. pettenkoferi* should influence clinical management. Retrospective studies have yet to confidently describe the potential pathogenicity of *S. pettenkoferi* [5]. However, multiple positive blood cultures across space and time, the indwelling catheter tip culture, and observed clinical improvement with appropriate treatment with antibiotics conferred strong evidence for true bacteremia with pathogenicity by *S. pettenkoferi*.

With the advent of MALDI-TOF mass spectrometry in clinical practice, it is suggested that *S. pettenkoferi* has been misidentified as other CoNS species due to similar morphology and biochemical results [6]. Therefore, there is anticipation that this species will be increasingly identified, and microbiologic studies have begun to demonstrate improved ability to differentiate S. pettenkoferi from other species [5, 7].

Hashi et al. [4] identified all reported cases of *S. pettenkoferi* true bacteremia from its first discovery in 2002 through 2015. These cases hailed from Germany [3], France [8], Belgium [9], South Korea [10], Brazil [11], Italy [12], Mexico [13], and Canada [4]. Subsequent literature review was performed on PubMed using the search terms “Staphylococcus pettenkoferi,” “case report,” “bacteremia,” “clinical,” and “United States.” Additional reported cases of true bacteremia were identified in France [14], South Korea [15], Russia [16], Poland [17], Italy [18], and Kenya [19].

In the United States, a ceftaroline susceptibility study [20] in American hospitals identified 1593 CoNS isolates for inclusion. Of this sample, ten cases were listed as infection by *S. pettenkoferi*. The study methodology states that inclusion in the surveillance program required isolates to be determined “[clinically] significant by local criteria as the reported probable cause of the infection.” However, supplemental data on the local criteria for clinical significance are lacking and the individual cases are not reported further.

Most recently, Eke et al. [5] identified 80 patient isolates of *S. pettenkoferi* at an American multi-site tertiary center from 2015 to 2019. Ninety percent of isolates were determined to be contaminates with the remaining 10% labeled as indeterminate. None met their criteria for true bacteremia, suggesting that the pathogenicity of *S. pettenkoferi* remains up for debate.

This case of true bacteremia by *S. pettenkoferi* is the first known report to originate from the United States. Immune deficiency has been described as a potential risk factor for clinical infection by *S. pettenkoferi*. Reported cases of bacteremia have been comorbid with extrapulmonary tuberculosis [3], diabetes [8], prematurity of the newborn [13], and AIDS [13]. In the case of our patient, both critical illness and treatment with anti-CD20 biologic agent rituximab conferred degrees of immune suppression.

Despite the lower virulence of CoNS in general, there is growing attention to the clinical relevance of CoNS bacteremia due to the consequences of indwelling device infection and treatment with broad-spectrum antibiotics. The ability to produce biofilms is a contributing mechanism of virulence for bacteria, and CoNS species, including *S. pettenkoferi*, are known to harbor biofilm-production genes [21, 22]. While two small studies were unable to replicate *S. pettenkoferi* biofilm production *in vitro* [6, 22], our patient's catheter tip culture yielded >15 colonies, supporting evidence for *in vivo* contamination and compromise of an indwelling device. For devices which cannot be removed readily or safely, such as intracardiac pacemaker wires or prosthetic valves, adherent bacterial colonies can prove more problematic. Additionally, due to other related antimicrobial-resistance genes, there are often limitations to deescalation of broad-spectrum antibiotics in treatment for CoNS. These genotypic and phenotypic features of CoNS species such as *S. pettenkoferi* are important considerations in patients with devices and immunocompromised status.

## 4. Conclusion

Infection by *S. pettenkoferi* is possibly underidentified and underreported. This case report joins a growing body of evidence for the potential pathogenicity of CoNS, in particular the relatively recently described species *S. pettenkoferi.*

## Figures and Tables

**Table 1:** Antimicrobial susceptibility testing results based on Microscan testing for *Staphylococcus pettenkoferi* isolate.

	Minimum inhibitory concentration (*μ*g/mL)	Interpretation
Clindamycin	<0.25	S
Erythromycin	1.0	I
Oxacillin	>2.0	R
Rifampin	≤1.0	S
Trimethoprim-sulfamethoxazole	≤0.5/9.5	S
Vancomycin	1.0	S
Tetracycline	≤4.0	S
Daptomycin	≤0.5	S

Abbreviations: S = susceptible; I = intermediate; R = resistant.

## Data Availability

The data used to support the findings of this study are presented in this case report; additional information from the case is available through the corresponding author upon request.
